# The Protective Effect of Transcription Factor 7-Like 2 Risk Allele rs7903146 against Elevated Fasting Plasma Triglyceride in Type 2 Diabetes: A Meta-Analysis

**DOI:** 10.1155/2015/468627

**Published:** 2015-10-04

**Authors:** Shuxia Wang, Kangxing Song, Roshni Srivastava, Mohsen Fathzadeh, Na Li, Arya Mani

**Affiliations:** ^1^The Geriatric Cardiology Department, Chinese PLA General Hospital, Beijing 100853, China; ^2^Yale Cardiovascular Research Center, Department of Internal Medicine, Yale University School of Medicine, New Haven, CT 06511, USA; ^3^Department of Cardiology, Chinese PLA General Hospital, No. 28, Fuxing Road, Beijing 100853, China; ^4^Department of Genetics, Yale University School of Medicine, New Haven, CT 06520, USA

## Abstract

*Background*. The results from published studies regarding association of transcription factor 7-like 2 (TCF7L2) variant rs7903146 with dyslipidemia have been conflicting and inconclusive. *Methods*. We carried out a meta-analysis that aimed to investigate the association of the rs7903146 variant with plasma lipid levels using electronic database and published studies. Data was extracted by a standard algorithm. Dominant, recessive, homozygote, and heterozygote comparison models were utilized. *Results*. 24 studies incorporating 52,785 subjects were included in this meta-analysis. Overall, the minor allele (T) was associated with lower risk for hypertriglyceridemia in subjects with type 2 diabetes (dominant model: SMD = −0.04, 95% CI (−0.08, 0.00), *P* = 0.048, *P*_heterogeneity_ = 0.47; recessive model: SMD = −0.10, 95% CI (−0.18, −0.02), *P* = 0.01, *P*_heterogeneity_ = 0.56). No association was found between minor (T) allele and plasma TC, LDL-c, or HDL-c levels in subjects with type 2 diabetes or metabolic syndrome (MetS) and no association was found between minor (T) allele and plasma TG levels in nondiabetic subjects. *Conclusions*. Our meta-analysis indicated the association between TCF7L2 rs7903146 polymorphism and low plasma triglyceride (TG) level in subjects with type 2 diabetes. No association was found between rs7903146 variant and plasma lipids in nondiabetic subjects.

## 1. Introduction

Dyslipidemia is a major risk factor for coronary heart disease and stroke [1, 2]. Despite extensive investigations, the underlying molecular mechanism of the disease in the general population is not understood [1]. Dyslipidemia is an inherited disorder with an estimated heritability of up to 60% for total cholesterol (TC) and HDL cholesterol (HDL-c) and 80% for plasma TG [2, 3]. With few exceptions, it is a complex trait caused by multiple genetic variations that together exert a sizeable effect on the trait [3].

The transcription factor 7-like 2 gene (TCF7L2), a Wnt signaling pathway effector, has been shown to be involved in the differentiation of adipocytes, regulation of adipokines, and pancreatic beta-cell function [4]. Polymorphisms of TCF7L2 have been identified as one of the most important genetic predictors of type 2 diabetes in genome-wide association studies [5]. The minor allele (T) of the TCF7L2 rs7903146 variant (C/T), located in intron 4 of TCF7L2, has been associated with increased risk for type 2 diabetes [6, 7]. In addition, several studies have suggested association between TCF7L2 rs7903146 polymorphism and dyslipidemia, but the results have been conflicting [8–11]. The objective of our meta-analysis was to investigate the association of the polymorphism rs7903146 of TCF7L2 with plasma lipids.

## 2. Methods

### 2.1. Literature Search

We systematically searched PubMed, Cochrane Library, Embase, reviews, and reference lists of relevant manuscripts before February 2014 by using Medical Subject Heading (MeSH) terms “Transcription Factor 7-like 2” or “TCF7L2” or “TCF7L” alone or paired with the following terms: “polymorphism,” “variant,” “SNP,” or “mutation.” The following four plasma lipids were included in our meta-analysis: TC, TG, LDL-c, and HDL-c.

### 2.2. Study Selection Criteria

The selection criteria for eligible studies were the following: (1) studies had to include data reported on at least one of the four fasting plasma lipids; (2) mean plasma lipid levels and standard deviations (SD) or standard errors were available; (3) studies included genotyping data; (4) study subjects were 18 years of age or older; (5) baseline data before intervention were available for interventional studies. Animal studies, case reports, review articles, abstracts, reports with incomplete data, and family-based studies were excluded.

### 2.3. Quality Assessment

The Newcastle-Ottawa Scale (NOS) was used to assess the quality of the studies. The NOS contains eight items categorized into three dimensions including selection, comparability, and exposure for case-control studies. In our meta-analysis, the NOS ranged from four to nine stars.

### 2.4. Data Extraction

Two investigators (Shuxia Wang and Kangxing Song) extracted data independently and discrepancies were resolved by consensus including a third author. Data were extracted using standardized methods. The following data were collected: article reference, first author, publication year, demographics including racial backgrounds, gender, age, health condition, and fasting status of the study subjects, mean and SD or standard error for the genotyping studies, genotyping methods, lipid assays, and unit of measurements.

### 2.5. Statistics and Analysis

Stata software (version 12.0, Stata Corporation, College Station, TX) and Review Manager 5.0 software (The Cochrane Collaboration, Oxford, UK) were used for the meta-analysis. Four different genetic models, including dominant model (TT + TC versus CC), recessive model (TT versus TC + CC), homozygote (TT versus CC), and heterozygote comparisons (TC versus CC), were used in our analysis. All data in this analysis were presented as mean ± SD. The SD was derived whenever the standard errors were reported. A pooled standardized mean difference (SMD) together with 95% confidence interval (CI) was used for this meta-analysis. SMD was selected to eliminate the effect of multiple scanners used for plasma lipid measurements [32]. A fixed-effect model was used for *P* values greater than 0.10 of the *χ* ^2^-based heterogeneity *Q*-tests, and random effect model was used for *P* values less than 0.10. The *I* ^2^ heterogeneity estimates were calculated and divided into 4 groups (*I* ^2^ = 0–25%, no heterogeneity; *I* ^2^ = 25–50%, moderate heterogeneity; *I* ^2^ = 50–75%, large heterogeneity; *I* ^2^ = 75–100%, extreme heterogeneity). The units of plasma lipids were converted from mmol/L to mg/dL, whenever necessary.

Funnel plots were used to evaluate publication bias. The funnel plots are asymmetric when there is publication bias. Egger's test, using MIX 1.7 software, was performed to estimate the degree of asymmetry of funnel plots. A stringent threshold of *P* < 0.1 was used as an indication for significant publication bias.

## 3. Results

### 3.1. Search Results

**Figure 1:**
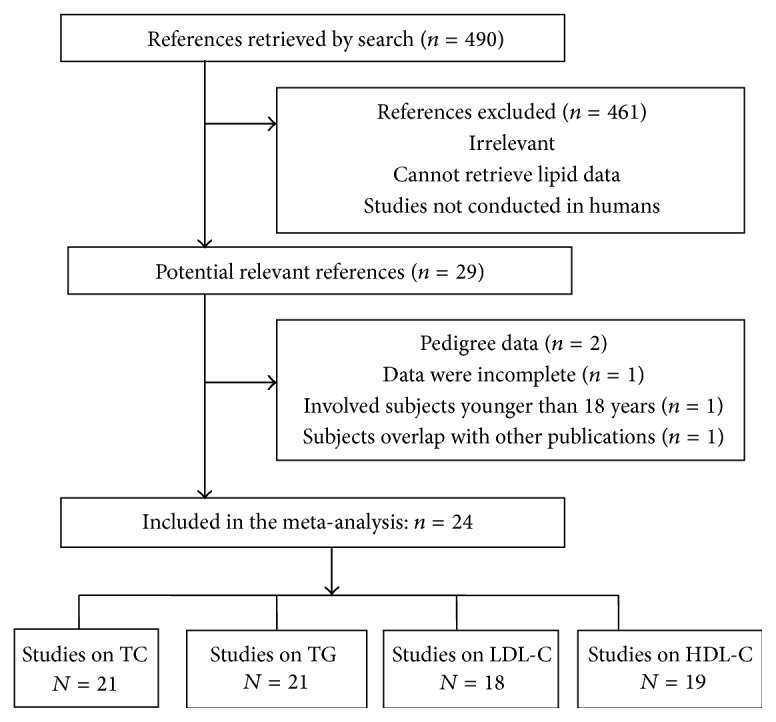
Flowchart outlining the process of search criteria and study selection.

A total of 490 articles were identified searching PubMed, Embase, and Cochrane Library databases and from a manual approach (search of previous studies cited in the reviews and the reference lists of identified articles); 461 articles were excluded because they were not conducted in human or did not meet the goals of this meta-analysis. Full text assessment of the 29 potentially relevant articles resulted in 24 eligible studies that met the inclusion criteria for the meta-analysis (Figure 1). The most common reasons for the exclusions were as follows: data were limited to isolated pedigrees [33, 34], were incomplete [35], or involved subjects younger than 18 years of age [36] and/or belonged to subjects that were studied in 2 or more published studies [37] (Figure 1).

A total of 24 studies [8–29, 31, 37] were included in the meta-analysis. The selected study characteristics are summarized in Table 1. There were 21, 21, 18, and 19 studies that reported plasma TC, TG, LDL-c, and HDL-c, respectively. Twenty-two studies included both men and women, one study [22] included only women, and one study [14] included only men. Within the eligible studies, eight [10, 12, 13, 15, 19, 26, 28, 31] involved subjects with diabetes, three [8, 21, 27] involved subjects with MetS, twelve [8, 10, 12–16, 20, 24, 25, 27, 28] involved nondiabetic healthy subjects, and eight [9, 11, 17, 18, 22, 23, 29, 37] involved general (community-based/population-based) subjects. Overall, 52,785 subjects were enrolled in this meta-analysis.

### 3.2. Association between rs7903146 and Plasma TG

**Figure 2:**
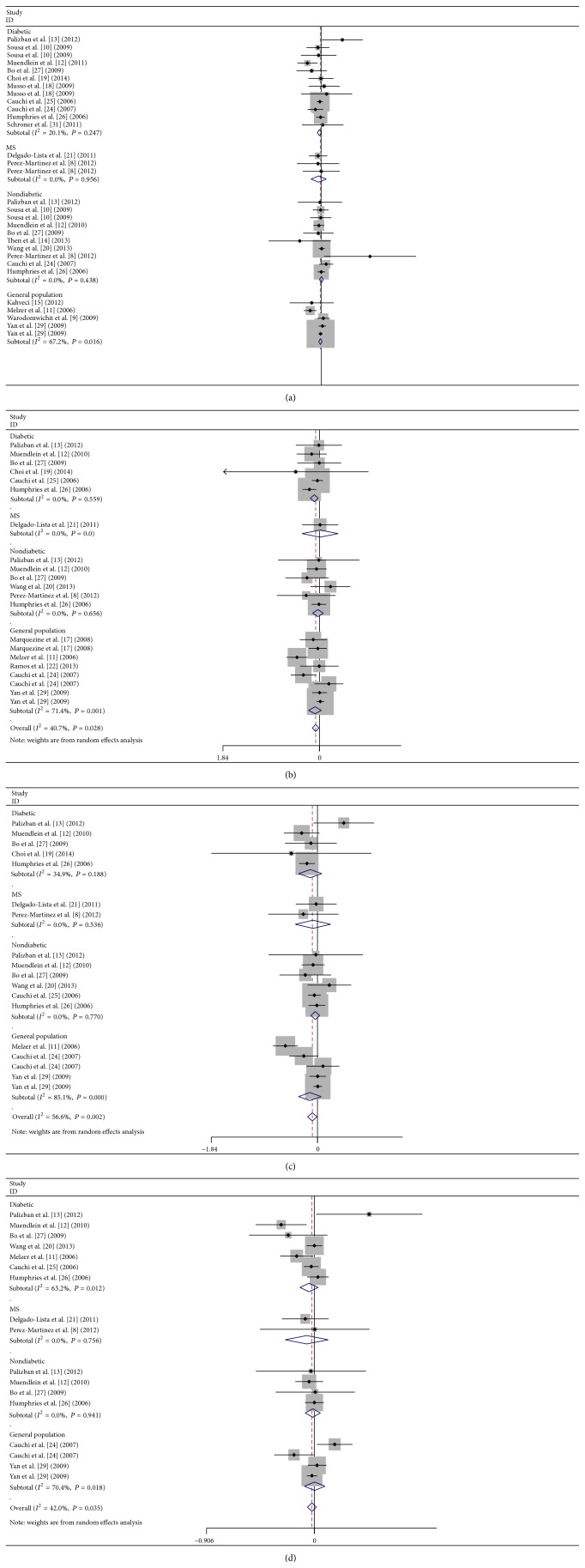
Forest plot of the TGCF7L2 rs7903146 polymorphism and triglyceride association: (a) dominant model, TT + TC versus CC, (b) recessive model, TT versus TC + CC, (c) homozygous model, TT versus CC, and (d) heterozygous model, TC versus CC.

The analysis under a dominant model on 30 comparisons showed that the carriers of T allele had tendency toward lower plasma TG than the noncarriers, but the results were not statistically significant: *P* = 0.07, SMD = −0.02, and 95% CI (−0.04, 0.00) (Figure 2(a)). No significant heterogeneity for this outcome was found (heterogeneity *χ* ^2^ = 36.54, *I* ^2^ = 21%, *P*_heterogeneity_ = 0.16) (Figure 2(a)). Under the recessive model, the carriers of TT genotype had significantly lower plasma TG than the noncarriers: *P* = 0.008, SMD = −0.05, and 95% CI (−0.09, −0.01) (Figure 2(b)). The heterogeneity for this outcome was significant (heterogeneity *χ* ^2^ = 33.65, *I* ^2^ = 41%, *P*_heterogeneity_ = 0.03) (Figure 2(b)). Under the homozygote comparison model, the carriers of TT genotype had lower plasma TG than CC genotype: *P* = 0.0086, SMD = −0.06, and 95% CI (−0.10, −0.02) (Figure 2(c)). The heterogeneity for this outcome was also significant (heterogeneity *χ* ^2^ = 38.98, *I* ^2^ = 56%, *P*_heterogeneity_ = 0.002) (Figure 2(c)). No difference was found in TG level between the carriers of TC genotype and CC genotype under heterozygote comparison model: *P* = 0.18, SMD = −0.02, and 95% CI (−0.04, 0.01) (Figure 2(d)). The heterogeneity for this outcome was also significant (heterogeneity *χ* ^2^ = 27.42, *I* ^2^ = 42%, *P*_heterogeneity_ = 0.02) (Figure 2(d)).

We then performed the subgroup analyses of the study population under four different genetic models.

Under dominant model, there was significant association between low plasma TG and T allele in diabetic subjects: *P* = 0.048, SMD = −0.04, 95% CI (−0.08, 0.00), *P*_heterogeneity_ = 0.47, but not in metabolic syndrome (MetS) subjects: *P* = 0.49, SMD = −0.06, 95% CI (−0.21, 0.10), *P*_heterogeneity_ = 0.96, or the nondiabetic subjects: *P* = 0.99, SMD = 0.00, 95% CI (−0.04, 0.04), *P*_heterogeneity_ = 0.48, or the general population: *P* = 0.27, SMD = −0.02, 95% CI (−0.05, 0.01), *P*_heterogeneity_ = 0.02 (Figure 2(a)).

Under a recessive model, there was significant association between low plasma TG and TT genotype in diabetic subjects: *P* = 0.01, SMD = −0.10, 95% CI (−0.18, −0.02), *P*_heterogeneity_ = 0.56 (Figure 2(b)), but not in nondiabetic subjects: *P* = 0.56, SMD = −0.03, 95% CI (−0.13, 0.07), *P*_heterogeneity_ = 0.66, or the general population: *P* = 0.15, SMD = −0.04, 95% CI (−0.09, 0.01), *P*_heterogeneity_ = 0.001 (Figure 2(b)).

Under a homozygote comparison model, there was no significant association between low plasma TG and TT versus CC genotype in any of the subgroups: diabetic subjects (*P* = 0.22, SMD = −0.13, 95% CI (−0.33, 0.08), *P*_heterogeneity_ = 0.19), MetS subjects (*P* = 0.62, SMD = −0.08, 95% CI (−0.39, 0.23), *P*_heterogeneity_ = 0.54), nondiabetic subjects (*P* = 0.32, SMD = −0.04, 95% CI (−0.12, 0.04), *P*_heterogeneity_ = 0.77), or general population (*P* = 0.19, SMD = −0.13, 95% CI (−0.33, 0.16), *P*_heterogeneity_ < 0.00001) (Figure 2(c)).

Under a heterozygote comparison model, there was no significant association between low plasma TG and TT/TC versus CC genotype in any of the subgroups: diabetic subjects (*P* = 0.25, SMD = −0.05, 95% CI (−0.12, 0.03), *P*_heterogeneity_ = 0.01) or MetS subjects (*P* = 0.48, SMD = −0.06, 95% CI (−0.25, 0.12), *P*_heterogeneity_ = 0.76), nondiabetic subjects (*P* = 0.68, SMD = −0.01, 95% CI (−0.08, 0.05), *P*_heterogeneity_ = 0.94), and general population (*P* = 0.99, SMD = 0.00, 95% CI (−0.08, 0.09), *P*_heterogeneity_ = 0.02) (Figure 2(d)).

### 3.3. Association between TCF7L2 rs7903146 Polymorphism and TC, HDL-c, and LDL-c

We performed the meta-analysis of the association of TCF7L2 rs7903146 polymorphism with TC, HDL-c, and LDL-c. There were no differences between rs7903146 polymorphism with TC, HDL-c, and LDL-c under 4 different genetic models (dominant, recessive, homozygote, and heterozygote) (Supplement Figures 1–3 in Supplementary Material available online at http://dx.doi.org/10.1155/2015/468627).

We then divided the study population into diabetic subjects, MetS subjects, nondiabetic subjects, and general populations using four different genetic models. No associations between rs7903146 polymorphism and TC, HDL-c, and LDL-c were detected in any of the subgroups under any of the four models (Supplement Figures 1–3).

### 3.4. Publication Bias

**Figure 3:**
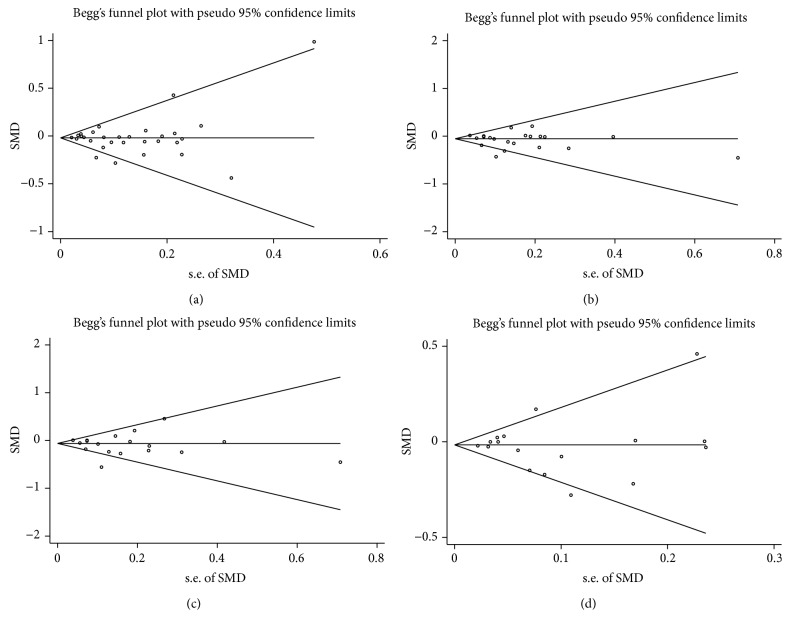
Funnel plot of TGCF7L2 rs7903146 polymorphism and triglyceride association in dominant model (a), recessive model (b), homozygous recessive model (c), and heterozygous model (d).

Publication bias was assayed by visual funnel plot inspection and Egger's test. The funnel plots comparing the differences in TC, TG, LDL-c, and HDL-c were all symmetric (Figures 3(a)–3(d) and Supplement Figures 4–6 A–D, resp.) and Egger's test did not indicate asymmetry of the plots.

## 4. Discussion

Our meta-analysis suggests that in subjects with type 2 diabetes or metabolic syndrome the minor TCF7L2 allele (rs7903146) has a protective effect against plasma TG but not TC, LDL-c, and HDL-c levels. There was no association between the minor allele and plasma lipids in nondiabetic subjects.

TCF7L2 rs7903146 is the most common susceptibility variant for type 2 diabetes across the world [6, 7]. The variant is associated with increased mRNA expression of TCF7L2 in pancreas [38, 39]. It increases the risk of type 2 diabetes by modifying the effect of incretins on insulin secretion [40], increasing gluconeogenesis and insulin resistance [41]. Accordingly, conditional deletion of TCF7L2 in adult hepatocytes results in reduced hepatic gluconeogenesis and lower plasma glucose levels [39]. Correspondingly, transient hepatic overexpression of TCF7L2 results in elevated plasma glucose levels [41]. Decreased insulin secretion and action are frequently associated with the simultaneous occurrence of hyperglycemia and dyslipidemia [42]. Therefore, at first sight, the result of our meta-analysis may appear as unexpected.

These results are, however, consistent with earlier findings in animal models. These studies have suggested that TCF7L2 may regulate plasma lipids through insulin-independent pathways. We have shown in LRP6 mutant mice that impaired Wnt signaling is associated with reduced TCF7L2 expression, increased de novo lipogenesis, and cholesterol synthesis and secretion in the liver, leading to high plasma TG levels. Reduced Wnt/TCF7L2 activity in these mice results in IGF1-dependent but insulin-independent activation of AKT-mTOR-SREBP axis [43]. Thus, increased TCF7L2 expression may have opposing effects on plasma glucose and plasma lipids. Alternatively, the protective effect of TCF7L2 minor variant against hypertriglyceridemia may be due to reduced adipose tissue lipolysis. In one earlier study, it was shown that reduced TCF7L2 in adipose tissue is associated with elevated triglyceride [34]. Finally, the minor allele in TCF7L2 may be in disequilibrium with other causal alleles in this gene. Regardless, these findings implicate TCF7L2 in regulation of plasma triglyceride in the general population and in conjunction with the animal studies identify this protein as a target for drugs against hyperlipidemia.

Heterogeneity was observed in some subgroups using certain models. In subgroup analysis for the association between risk allele and TG, heterogeneity was found in diabetic subjects under heterozygote model and in general subjects under all four different genetic models. For the analysis of association between LDL and the risk allele, the heterogeneity was only found under dominant model in subjects with type 2 diabetes and MetS subjects. The association analysis of HDL with risk allele showed heterogeneity for subjects with type 2 diabetes and general subjects under dominant, recessive, and heterozygote models. There was no heterogeneity in subgroup analysis of TC with risk allele. Random effect models were used to decrease the effect of heterogeneity in subgroup analysis. The present analysis had several limitations. First, most of the included studies were designed to study the association of TCF7L2 variants with diabetes [11, 13, 15–17, 19, 20, 26, 28, 29, 31], hyperglycemia [23, 25], and coronary artery disease [10] and only 2 studies [8, 9] were exclusively designed to examine the association between rs7903146 and plasma lipid levels. For all other studies, the lipid levels had to be extracted from baseline characteristics of the study populations. These studies were not designed to introduce the necessary adjustments for confounding factors such as lifestyle changes and medications [27]. Nonetheless, the effects of such variables were significantly reduced in our analysis by using preintervention baseline data. Second, the meta-analysis was based on unadjusted estimates. In absence of detailed demographics, a post hoc analysis of adjusted estimates could not be carried out. One other limitation is related to inadequate data on MetS. There were only two publications that specifically studied subjects with MetS. Consequently, no analyses could be carried out for this subgroup.

In conclusion, our meta-analysis identified an association between T genotype of TCF7L2 rs7903146 with lower TG level in subjects with diabetes. This finding identifies TCF7L2 as a potential target for development of novel therapeutics against hyperlipidemia.

## Supplementary Material

Supplement Figures 1-3 Forest plots of the TGCF7L2 rs7903146 polymorphism and total cholesterol, LDL-cholesterol, and HDL-cholesterol association. Under different models: A: dominant.
Model, TT+TC vs. CC; B: recessive model, TT vs. TC+CC; C: Homozygous, TT vs. CC; D: Heterozygous, TC vs. CC.
Supplement Figure 4: Funnel plot of TGCF7L2 rs7903146 polymorphism and total cholesterol, LDL-cholesterol and association under different models: dominant model (A) recessive model (B), homozygous recessive model (C) and heterozygous model (D).

## Figures and Tables

**Table 1:** Characteristics of the study populations in the included studies.

First author, year, and reference	Ethnicity	Gender	Age	Study population	Outcome
Sousa 2009 [10]	Brazil	M/F	58.4–62.7	Nondiabetic subjects, diabetic subject	TC, LDL-c, HDL-c, TG
Muendlein 2011 [12]	Austria	M/F	63.3–65.9	Diabetic subject, nondiabetic subjects	TC, LDL-c, HDL-c, TG
Palizban 2012 [13]	Iran	M/F	50.4–59.3	Diabetic subject, nondiabetic subjects	TC, TG
Then 2013 [14]	German	M	51–58	Nondiabetic subjects	TC, LDL-c, HDL-c, TG
Kahveci 2012 [15]	Turkey	M/F	36–84	Diabetic subject and nondiabetic subjects	TC, LDL-c, HDL-c, TG
Bodhini 2007 [16]	Asian Indians	M/F	41 ± 11	Normal glucose tolerant subjects	TC, LDL-c, HDL-c, TG
Warodomwichit 2009 [9]	European Americans	M/F	17–92 ys	The Genetics of Lipid Lowering Drugs and Diet Network (GOLDN) study	TC, LDL-c, HDL-c, TG
Melzer 2006 [11]	White European origin	M/F	≥65 ys	Older population from InCHIATI study	TC, LDL-c, HDL-c, TG
Marquezine 2008 [17]	Brazil	M/F	44.3–59.8	Multivessel coronary artery disease patients, general population	TC, HDL-c, TG
Musso 2009 [18]	Italy	M/F	38–41	Healthy control, nonalcoholic fatty liver disease	TC, LDL-c, HDL-c, TG
Choi 2014 [19]	Korea	M/F	59-60	Diabetic patients	TC, LDL-c, HDL-c, TG
Wang 2013 [20]	Chinese	M/F	20–85	Nondiabetic controls	TC, LDL-c, HDL-c, TG
Delgado-Lista 2011 [21]	LIPGENE dietary intervention from 8 European countries	M/F	35–70 ys	Metabolic syndrome according to NCEP criteria	TC, LDL-c, HDL-c, TG
Perez-Martinez 2012 [8]	Ireland	M/F	22–68.4	Young men, metabolic syndrome, and healthy elderly	TC, LDL-c, HDL-c, TG
Ramos 2013 [22]	Brazil	F	22.8 ± 6.6	Women with polycystic ovary syndrome	TC, LDL-c, HDL-c, TG
Gambino 2010 [23]	Caucasian, Italy	M/F	54.2–54.7	Population-based cohort	LDL-c, HDL-c
Cauchi 2007 [24]	Caucasian, French	M/F	Around 22	Small gestational age, appropriate for gestational age birth weight	TC, TG
Cauchi 2006 [25]	French	M/F	30–65	Normoglycemic	TC, LDL-c, HDL-c, TG
Humphries 2006 [26]	European White, Indian Asia, Afro-Caribbean	M/F	62.4–63	Diabetic patients, healthy subjects	TC, LDL-c, HDL-c, TG
Bo 2009 [27]	Italy	M/F	55.2–56.8	Nondiabetic dysmetabolic participants, control	HDL-c, TG
Ereqat 2010 [28]	Palestinian	M/F	>40 ys	Diabetic subject, nondiabetic subjects	TC
Yan 2009 [29]	African American, Caucasian	M/F	53-54	The Atherosclerosis Risk in Communities Study (ARIC)	LDL-c, HDL-c, TG
Yan 2010 [30]	African American, Caucasian	M/F	58.3–60.1	The Atherosclerosis Risk in Communities Study (ARIC)	TC
Schroner 2011 [31]	Slovakia	M/F	62.6–63.1	Diabetic subject with HbA1C <7.0%	TC, LDL-c, HDL-c, TG
