# Supplementary material for: The Protective Effect of Transcription Factor 7-Like 2 Risk Allele rs7903146 against Elevated Fasting Plasma Triglyceride in Type 2 Diabetes: A Meta-Analysis
Source: J Diabetes Res. 2015 Oct 4;2015:468627. doi: 10.1155/2015/468627 (PMC4631899; doi:10.1155/2015/468627)
Supplement: Supplementary file 1 — Supplement Figures 1-3 Forest plots of the TGCF7L2 rs7903146 polymorphism and total cholesterol, LDL-cholesterol, and HDL-cholesterol association. Under different models: A: dominant. Model, TT+TC vs. CC; B: recessive model, TT vs. TC+CC; C: Homozygous, TT vs. CC; D: Heterozygous, TC vs. CC. Supplement Figure 4: Funnel plot of TGCF7L2 rs7903146 polymorphism and total cholesterol, LDL-cholesterol and association under different models: dominant model (A) recessive model (B), homozygous recessive model (C) and heterozygous model (D). [file 468627.f1.pdf]

Supplemental Figure 1

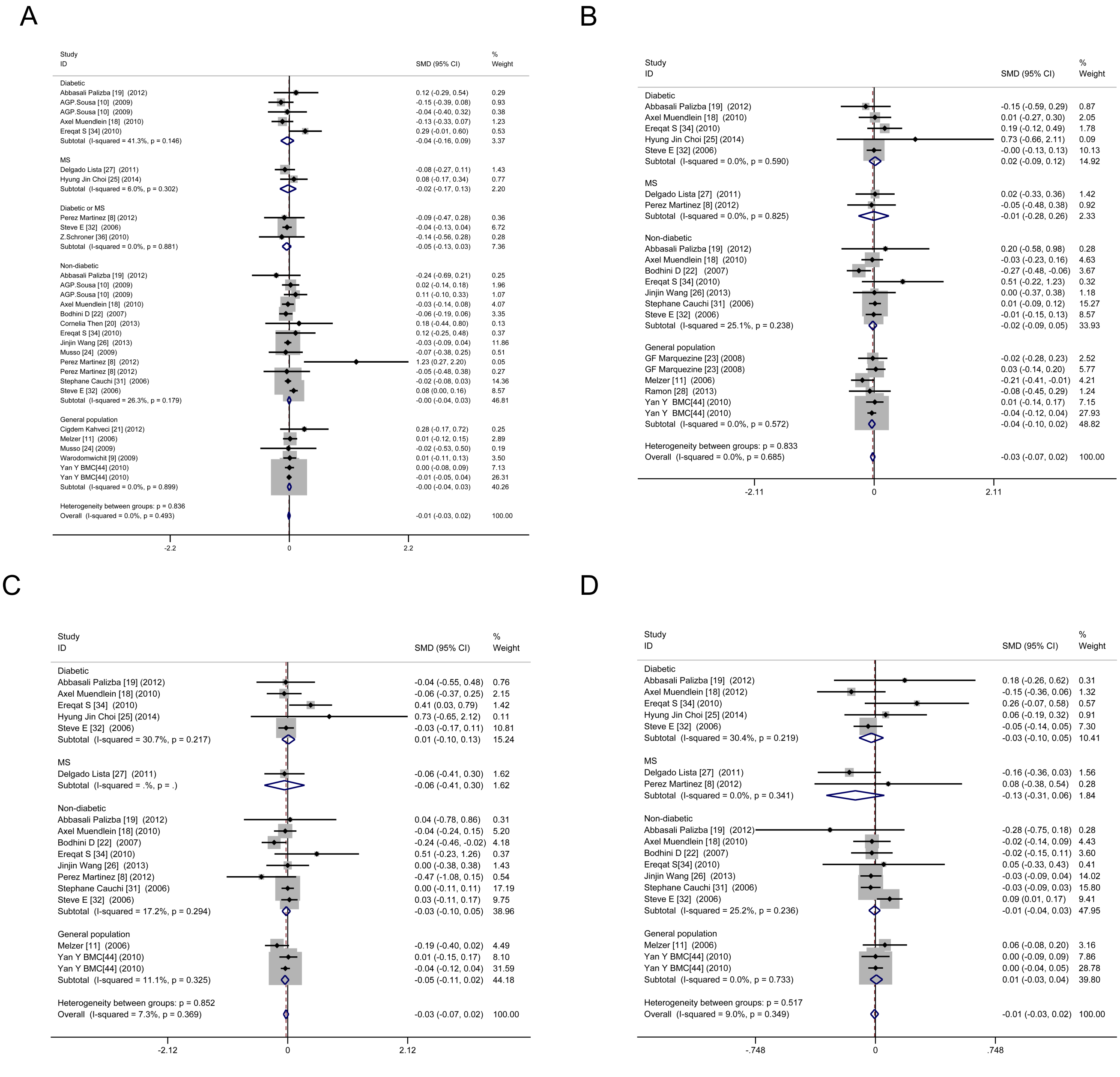

Forest plot of the TGCF7L2 rs7903146 polymorphism and cholesterol association. A: dominant Model, TT+TC vs CC; B: recessive model, TT vs TC+CC; C:Homozygous, TT vs CC; D: Heterozygous, TC vs CC.

Supplemental Figure 2

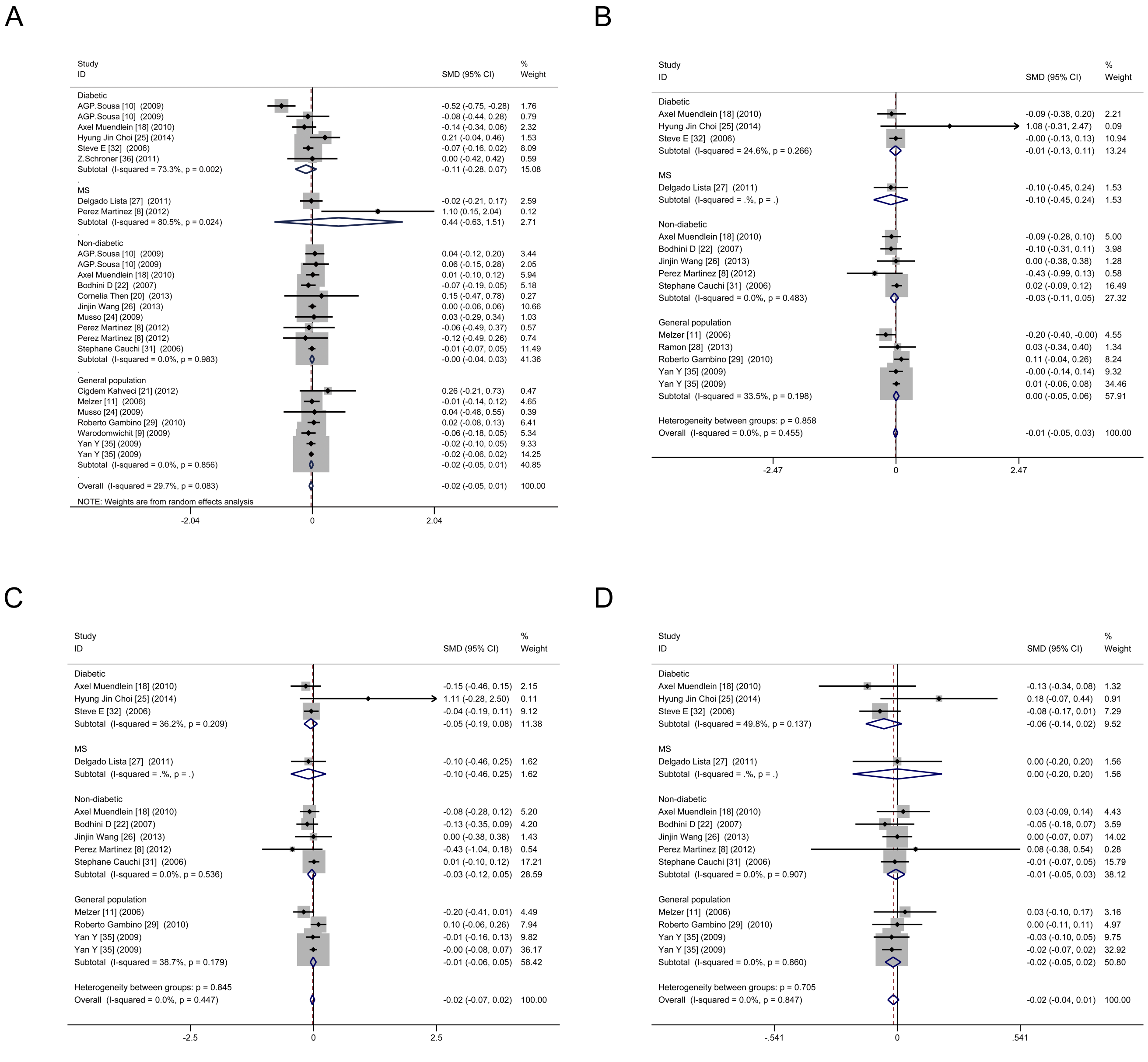

Forest plot of the TGCF7L2 rs7903146 polymorphism and LDL-cholesterol association. A: dominant Model, TT+TC vs CC; B: recessive model, TT vs TC+CC; C:Homozygous, TT vs CC; D: Heterozygous, TC vs CC.

Supplemental Figure 3

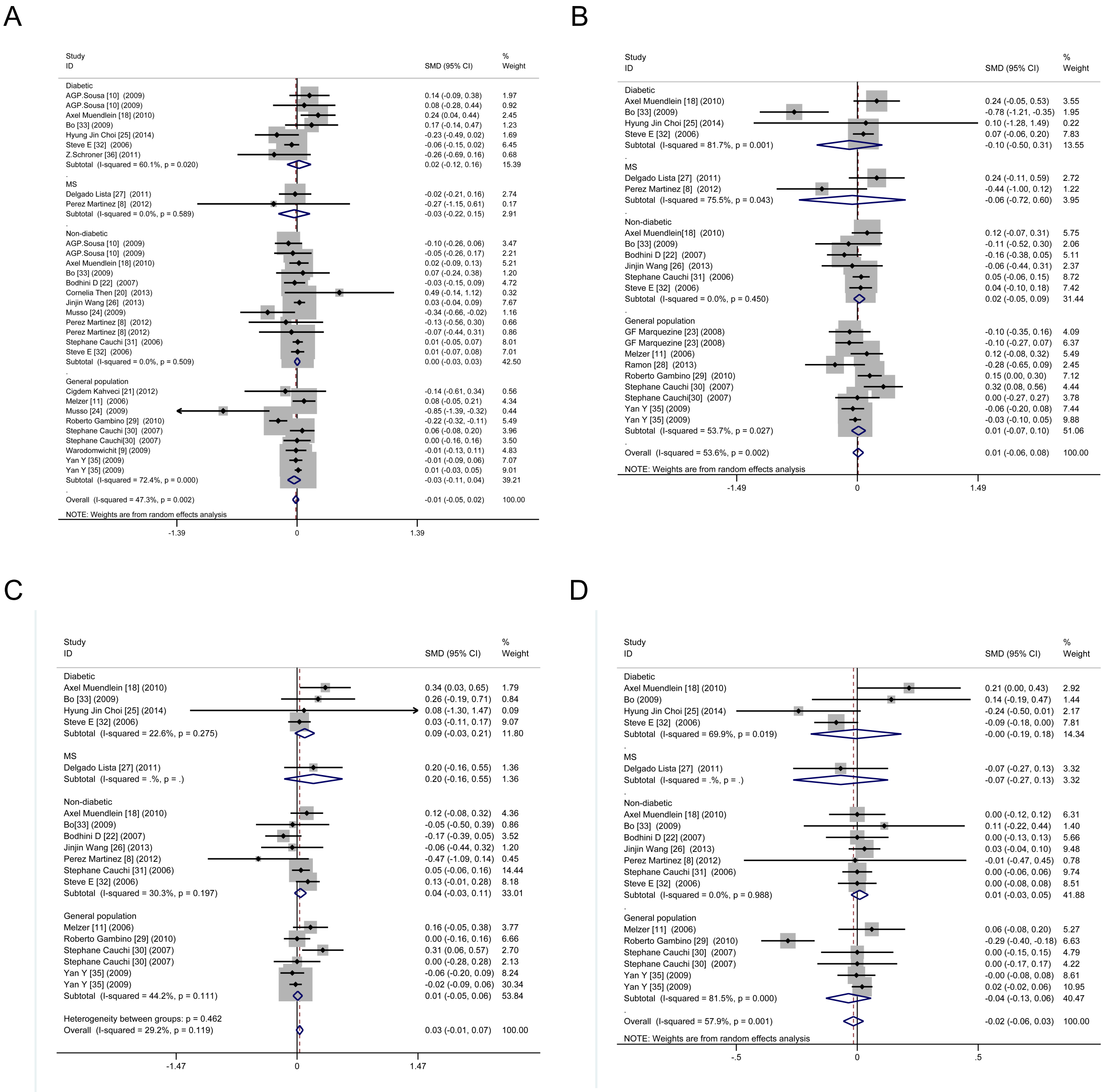

Forest plot of the TGCF7L2 rs7903146 polymorphism and HDL-cholesterol association. A: dominant Model, TT+TC vs CC; B: recessive model, TT vs TC+CC; C:Homozygous, TT vs CC; D: Heterozygous, TC vs CC.

Supplemental Figure 4

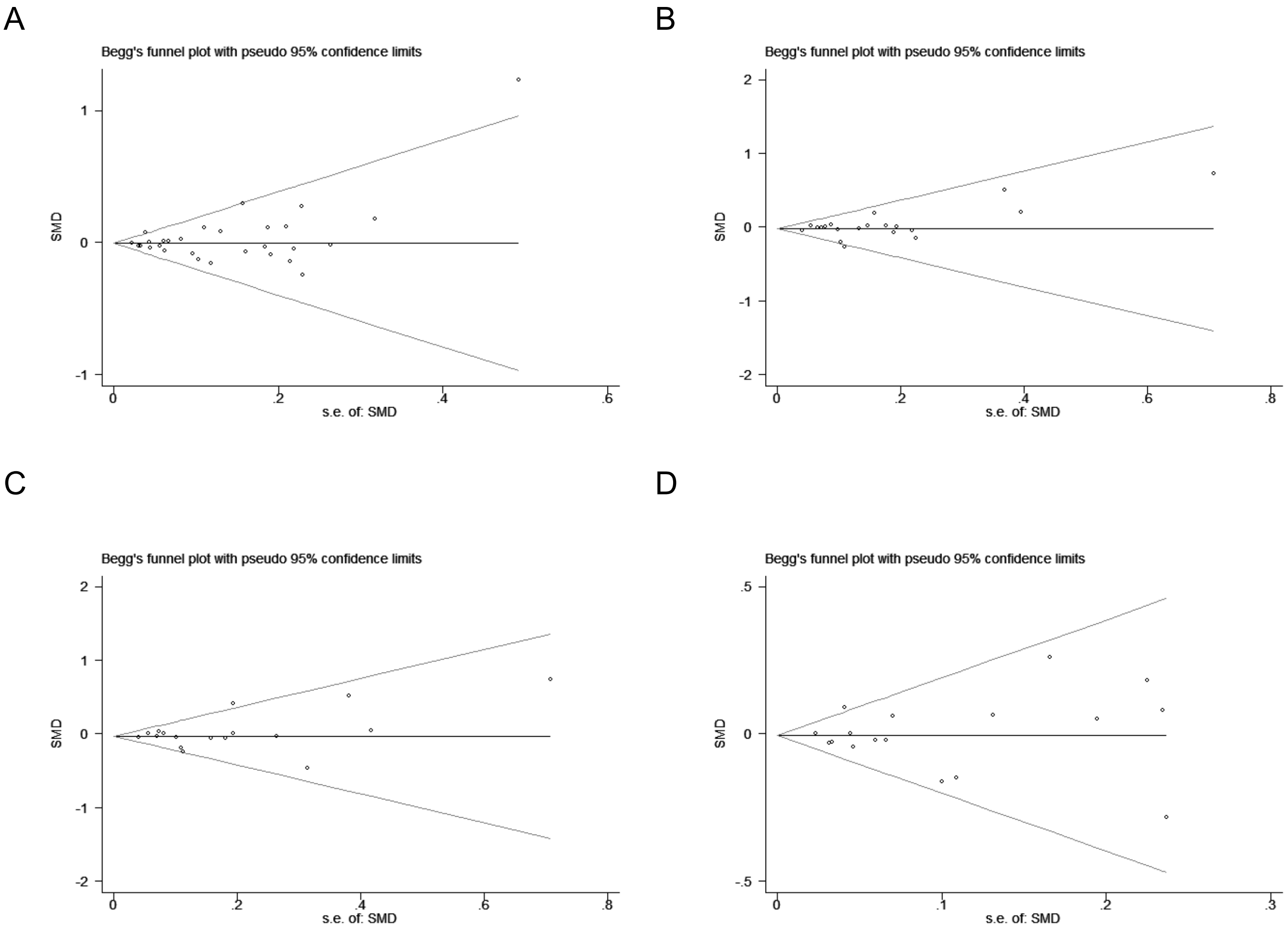

Funnel plot of TGCF7L2 rs7903146 polymorphism and cholesterol association in dominant model (A), recessive model (B), homozygous recessive model (C) and heterozygous model (D)

Supplemental Figure 5

A

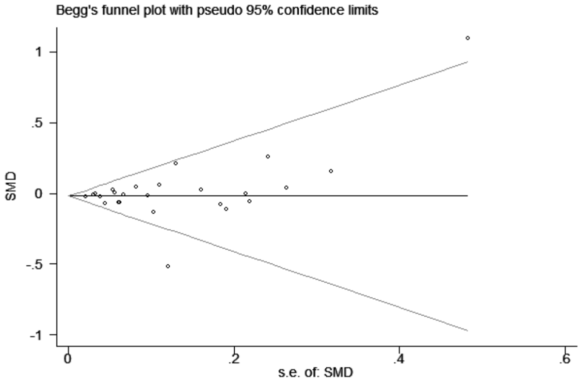

B

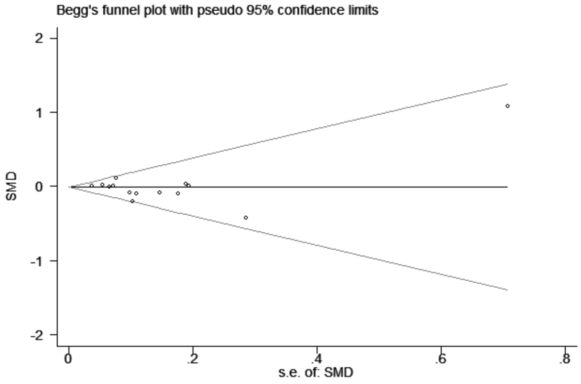

C

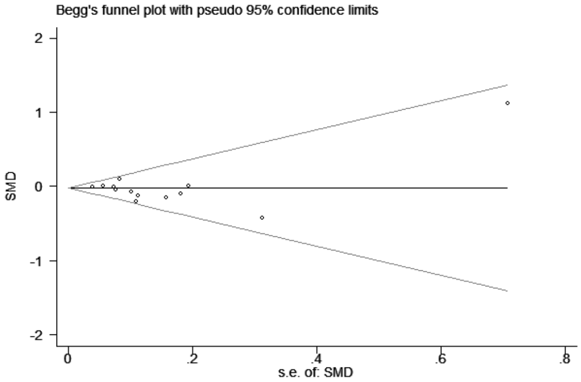

D

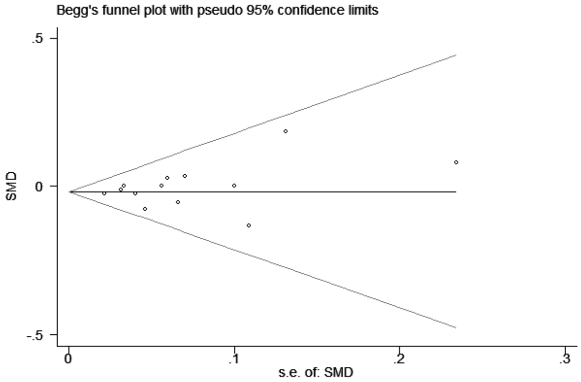

Funnel plot of TGCF7L2 rs7903146 polymorphism and LDL-cholesterol association in dominant model (A), recessive model (B), homozygous recessive model (C) and heterozygous model (D).

Supplemental Figure 6

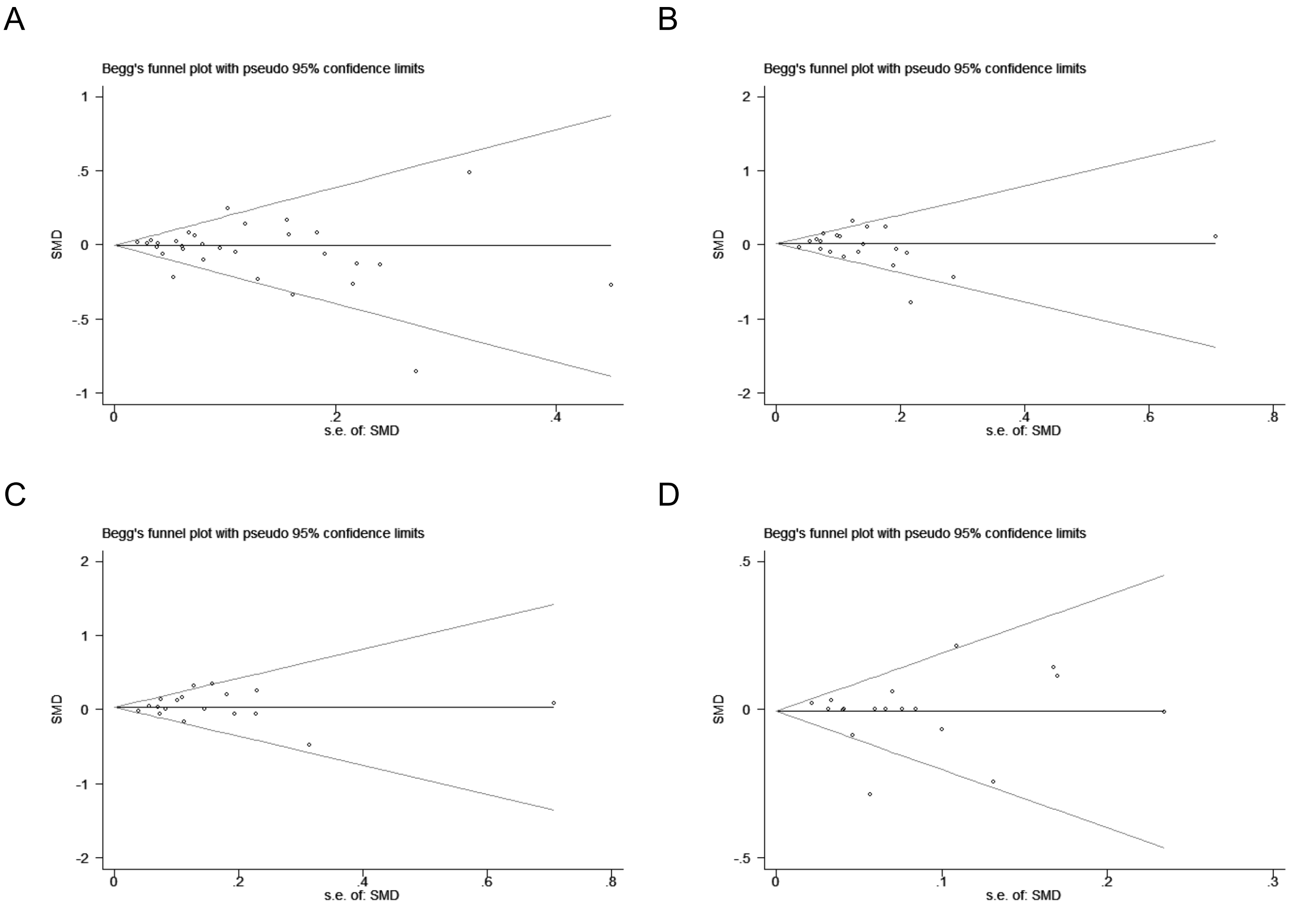

Funnel plot of TGCF7L2 rs7903146 polymorphism and HDL-cholesterol association in dominant model (A), recessive model (B), homozygous recessive model (C) and heterozygous model (D).
